# Can preventive care activities in general practice be sustained when financial incentives and external audit plus feedback are removed? *ACCEPt-able*: a cluster randomised controlled trial protocol

**DOI:** 10.1186/s13012-016-0489-0

**Published:** 2016-09-13

**Authors:** Jane S. Hocking, Meredith Temple-Smith, Mieke van Driel, Matthew Law, Rebecca Guy, Liliana Bulfone, Anna Wood, Nicola Low, Basil Donovan, Christopher K. Fairley, John Kaldor, Jane Gunn

**Affiliations:** 1 Melbourne School of Population and Global Health, University of Melbourne, 3/207 Bouverie Street, Carlton, 3053 VIC Australia; 2 Department of General Practice, University of Melbourne, Melbourne, Australia; 3 Discipline of General Practice, University of Queensland, Brisbane, Australia; 4 Kirby Institute, UNSW Australia, Sydney, Australia; 5 Deakin Health Economics, Deakin University, Melbourne, Australia; 6 University of Bern, Bern, Switzerland; 7 Melbourne Sexual Health Centre, Monash University, Melbourne, Australia

**Keywords:** Financial incentives, Audit and feedback, Cluster randomised controlled trial, Primary care, Preventive care

## Abstract

**Background:**

Financial incentives and audit plus feedback on performance are two strategies commonly used by governments to motivate general practitioners (GP) to undertake specific healthcare activities. However, in recent years, governments have reduced or removed incentive payments without evidence of the potential impact on GP behaviour and patient outcomes. This trial (known as *ACCEPt-able*) aims to determine whether preventive care activities in general practice are sustained when financial incentives and/or external audit plus feedback on preventive care activities are removed. The activity investigated is annual chlamydia testing for 16- to 29-year-old adults, a key preventive health strategy within this age group.

**Methods/design:**

ACCEPt-able builds on a large cluster randomised controlled trial (RCT) that evaluated a 3-year chlamydia testing intervention in general practice. GPs were provided with a support package to facilitate annual chlamydia testing of all sexually active 16- to 29-year-old patients. This package included financial incentive payments to the GP for each chlamydia test conducted and external audit plus feedback on each GP’s chlamydia testing rates. ACCEPt-able is a factorial cluster RCT in which general practices are randomised to one of four groups: (i) removal of audit plus feedback—continue to receive financial incentive payments for each chlamydia test; (ii) removal of financial incentive payments—continue to receive audit plus feedback; (iii) removal of financial incentive payments and audit plus feedback; and (iv) continue financial incentive payments and audit plus feedback. The primary outcome is chlamydia testing rate measured as the proportion of sexually active 16- to 29-year-olds who have a GP consultation within a 12-month period and at least one chlamydia test.

**Discussion:**

This will be the first RCT to examine the impact of removal of financial incentive payments and audit plus feedback on the chlamydia testing behaviour of GPs. This trial is particularly timely and will increase our understanding about the impact of financial incentives and audit plus feedback on GP behaviour when governments are looking for opportunities to control healthcare budgets and maximise clinical outcomes for money spent. The results of this trial will have implications for supporting preventive health measures beyond the content area of chlamydia.

**Trial registration:**

The trial has been registered on the Australian and New Zealand Clinical Trials Registry (ACTRN12614000595617).

## Background

Primary health care plays a pivotal role in preventive health, and many different strategies have been employed to support healthcare professionals to undertake preventive health activities with their patients. Financial incentives and audit plus feedback on performance are two common strategies, used with varying levels of success [1]. Financial incentives have been a popular approach to increase efficiency in health care, and pay for performance schemes (P4P) have been widely implemented throughout the United States of America (USA), United Kingdom (UK), Canada and Australia [2, 3]. In P4P, healthcare providers receive explicit financial incentives based on their performance related to preventive activities undertaken, clinical quality, resource use and patient-reported outcomes. Audit and feedback is another intervention widely used to improve professional practice and is part of ongoing professional development programmes for general practitioners (GPs) in Australia, the UK and elsewhere [4–6].

In Australia, GPs are the point of entry to primary healthcare services with over 85 % of Australians attending general practice each year [7]. The healthcare system in the UK is similar, and financial incentives are used in both countries to promote evidence-based practice and encourage a greater output of health prevention activities. In Australia, financial incentives in primary care are provided through the Practice Incentives Program (PIP) which was introduced in 1998 [8]. Financial incentives are paid to the general practice for 11 activities and include incentives for quality prescribing, early diagnosis and effective management of diabetes and continuing care for patients with asthma [9]. Within the PIP framework, the Service Incentives Payment (SIP), introduced in 2001, provides the possibility for additional payments made directly to GPs for completing “cycles of care” for patients with diabetes and asthma and also for conducting cervical screening of under-screened women. At $AU 282 million per year, this financial incentive payment scheme is a significant cost to the Australian government [10]. In the UK, the quality and outcomes framework (QOF) was introduced in 2004 and is arguably the most comprehensive national primary care P4P scheme in the world [11, 12]. UK general practice is remunerated according to performance against different indicators including clinical care (e.g. diabetes), organisational issues (e.g. management of medicines and summarisation of patients’ records) and patients’ experiences [12, 13]. This UK scheme costs over £1 billion per year to administer [14].

Audit and feedback is another intervention widely used to improve professional practice and is part of ongoing professional development programmes for GPs in Australia, UK and elsewhere [4, 5, 15, 16]. In an audit plus feedback process, an individual’s professional practice is measured and then compared to professional standards or targets and/or their peers. The results are then given back to the individual. The expectation is that healthcare professionals are prompted to modify their practice if given feedback that their clinical practice was inconsistent with that of their peers or accepted guidelines.

In an effort to reduce health budgets, recent changes have seen a reduction in PIP payments in Australia. Incentives for childhood immunisation and after-hours care availability items have been removed, and the threshold targets for payment eligibility have been increased for the cervical cancer screening and diabetes management indicators [9]. In the UK, there have also been changes to the QOF with further increases in the threshold targets for payment eligibility leading to reduced GP income [17]. By building on an existing trial of a chlamydia testing intervention in general practice (the Australian Chlamydia Control Effectiveness Pilot [ACCEPt]), we have the unique opportunity to provide the first randomised controlled trial (RCT) evidence about the impact of removing financial incentives and audit and feedback on the preventive healthcare activities of GPs in Australia. ACCEPt included financial incentive payments per chlamydia test ordered and audit and feedback on chlamydia testing performance for each GP in the intervention arm of this 3-year chlamydia testing trial. We will re-randomise intervention general practices at the conclusion of ACCEPt to investigate the impact of removing financial incentive payments and/or audit plus feedback on GPs’ chlamydia testing rates, a key preventive healthcare activity for young adults [18–20]. This new trial, referred to as *ACCEPt-able*, is described below.

### Aims and objectives

ACCEPt-able aims to determine whether preventive care activities in general practice are sustained when financial incentive payments and/or external audit plus feedback on preventive care activities are removed.

The trial objectives are

1. To investigate the impact of removing financial incentives on preventive care activities in general practice, following implementation of a preventive care intervention
2. To investigate the impact of removing external audit plus feedback on preventive care activities in general practice, following implementation of a preventive care intervention
3. To evaluate the views and acceptability of financial incentive payments and external audit plus feedback among GPs and other clinic staff
4. To conduct a cost-consequence analysis (a form of economic analysis that compares alternative interventions) comparing scenarios for continuation/discontinuation of financial incentive payments and external audit plus feedback activities

## Methods

### Setting for ACCEPt-able

ACCEPt-able builds on the ACCEPt RCT conducted in Australian general practice. ACCEPt evaluated whether a multifaceted intervention could increase chlamydia testing and reduce the prevalence of chlamydia among sexually active 16- to 29-year-old men and women attending general practice. Chlamydia is the most common bacterial sexually transmitted infection (STI) worldwide with over 100 million men and women infected at any point in time [21]. Annual chlamydia screening among under 30-year-old adults is a key preventive care activity [18].

### Design of ACCEPt

ACCEPt was evaluated using a cluster RCT design (Australian New Zealand Clinical Trials Register, ACTRN12610000297022) [22]. An intervention to support increased chlamydia testing was allocated at the geographical area (postcode) level in four Australian states (New South Wales, Victoria, Queensland and South Australia) and all general practices in each postcode participated. GPs in the intervention group were asked to offer an annual chlamydia test to all sexually active 16- to 29-year-old patients, and they received a multifaceted support package designed to facilitate chlamydia testing. Each practice in the intervention group received the following: (i) financial incentive payments to the GP for each chlamydia test conducted. These payments ranged from $AU 5 per eligible test for up to 20 % coverage to $AU 8 per test for over 40 % coverage. These payments were reimbursed to the GP every 3 months. The amount of these payments was based on similar incentives available to GPs for immunisations in Australia at the time the trial commenced in 2010. (ii) Audit and feedback by provision of quarterly chlamydia testing reports for each GP and the general practice. The report listed the number of patients aged 16 to 29 years that the GP tested during the quarter, his/her chlamydia testing rate (numerator = total number of chlamydia tests done for 16- to 29-year-olds; denominator = total number of patients aged 16 to 29 years seen at least once for a consultation during the time period) for the quarter and for the previous 12 months. The feedback reports were provided in person every 3 months by a research officer. Further detail can be found in the ACCEPt protocol [22].

ACCEPt was completed in December 2015 with a total of 125 general practices (63 intervention and 62 control) participating. Annual testing rates in the intervention group increased from 8.1 % in the year prior to the trial to 19.8 % in the final year of the trial. Two general practices withdrew from ACCEPt; both were in the control group.

### Design of ACCEPt-able

ACCEPt-able is a factorial cluster RCT (Australian and New Zealand Clinical Trials Registry ACTRN 1261400059617) with the general practice being the unit of randomisation. An RCT has a factorial design when two or more experimental interventions are not only evaluated separately but also in combination and against a control [23]. The strength of this design is that it provides more information than parallel designs. Our intervention is allocated at the cluster level (general practice) because patients attending each practice can consult with multiple different GPs over time but they are only eligible for one chlamydia test each year unless they report additional risk factors for infection (e.g. recent change in sex partner) or genital symptoms requiring further chlamydia testing.

**Fig. 1:**
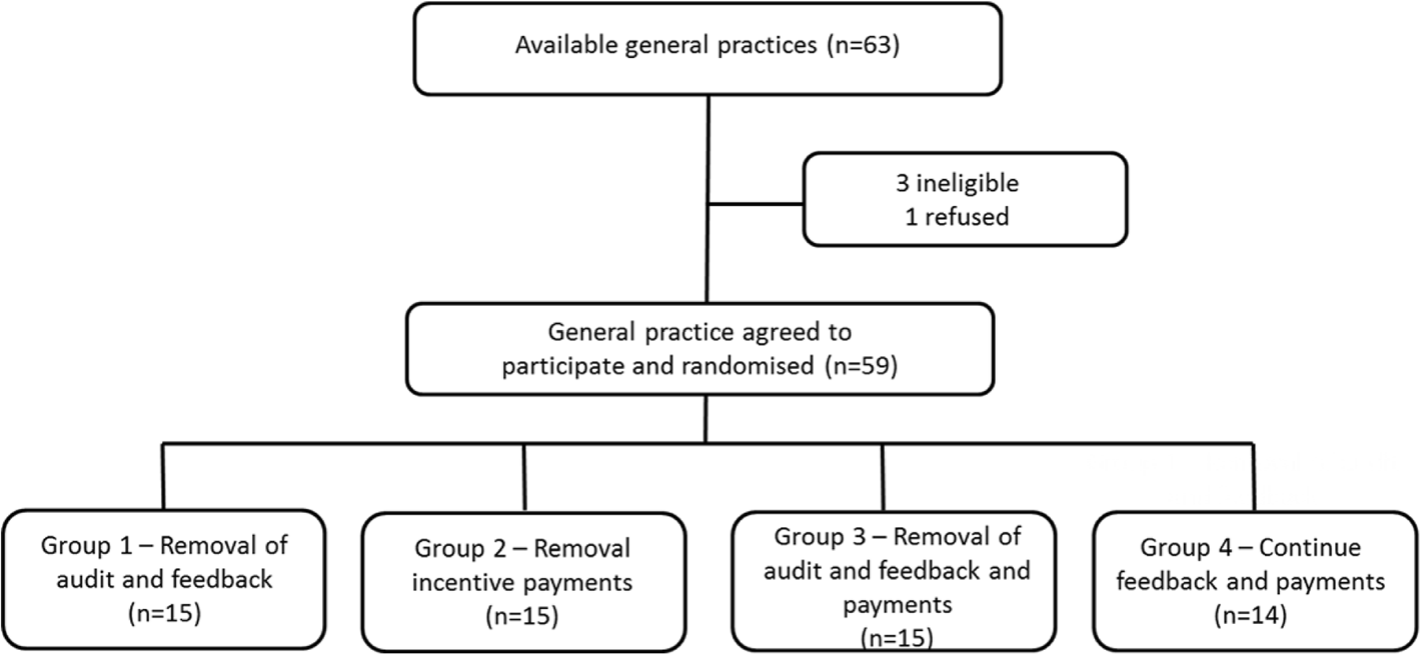
*ACCEPt-able* flow chart

Practices were recruited into ACCEPt-able when their participation in ACCEPt was completed. A research officer explained the trial to the GPs and once the GPs consented to participate, the practices were randomised to one of four groups. (Fig. 1).

### Duration

The duration of the intervention period for ACCEPt-able will be up to 2 years. Recruitment commenced in September 2014 and was completed in September 2015. The trial is due to be completed in late 2017.

### Inclusion criteria

To be eligible to participate in ACCEPt-able, general practices must have been participating in ACCEPt, have been in the intervention group and they must have GRHANITE™, a data extraction tool (see below for further information) installed on their medical records software.

### Intervention

General practices participating in ACCEPt-able were re-randomised into one of the following four trial groups:

1. Removal of audit and feedback—continue incentive payments only (group 1): GPs and general practices in this group continue to receive incentive payments for each test conducted as described above.
2. Removal of financial incentive payments—continue audit plus feedback only (group 2): each GP will continue to receive a report outlining their chlamydia testing performance over the last quarter as described above.
3. Removal of financial incentive payments and audit plus feedback (group 3): GPs and general practice staff will not receive any feedback or incentive payments for chlamydia tests undertaken during the trial.
4. Continue financial incentive payments and audit plus feedback (group 4): GPs and general practices will continue to receive both quarterly feedback and quarterly incentive payments for all chlamydia tests done for eligible patients.

### Randomisation, allocation concealment and blinding

The unit of randomisation (cluster) is the general practice, and these have been randomised using a minimisation approach that maximises the balance across two baseline variables. These baseline variables are annual chlamydia testing rates among 16- to 29-year-old men and women for the 12-month period prior to commencing ACCEPt-able and size of the practice (number of patients seen each year). Practices have been progressively randomised into ACCEPt-able once their participation in ACCEPt is complete. The trial statistician is located at a site away from any of the participating general practices. The statistician allocated each general practice according to the computer-generated algorithm and informed research staff of the randomisation outcome for each practice. Blinding of general practices and GPs to their trial allocation is not possible given the nature of the intervention.

### Outcomes

The primary outcome is the annual chlamydia testing rate measured as the proportion of 16- to 29-year-olds consulting the general practice for any reason who have at least one chlamydia test in a 12-month period. The numerator is the number of people aged 16 to 29 years who have at least one chlamydia test within a 12-month period; the denominator is the number of people aged 16 to 29 years who have at least one GP consultation during the same 12-month period.

### Data collection

We have installed GRHANITE™ [24], a data extraction tool developed by the University of Melbourne, at all general practices participating in ACCEPt-able. The tool extracts anonymous data about all consultations including age and gender of the patient, a unique non-identifying patient code that enables us to track repeat visits to any participating general practice, chlamydia pathology test ordering, and test results. GRHANITE™ encrypts data on the general practice computer and electronically exports them to a secure database at the University of Melbourne once per week. These data are used to calculate chlamydia testing rates for participating GPs. In addition, we collect data for factors that might confound associations with our outcome of chlamydia testing rates. These data include the individual’s age and gender and GP’s years of experience in general practice, as well as area level data such as the size of the underlying population and the socioeconomic profile of the area according to the Australian Bureau of Statistics Index of Relative Disadvantage.

### Sample size

The intra-cluster correlation for chlamydia testing in participating general practices in the 12 months prior to commencing ACCEPt-able was 0.02. The cluster size of an average of 700 patients aged 16 to 29 years seen in each participating general practice per year gives a design effect of 15. With 60 general practices available for randomisation and an average of 700 patients per practice, we get an effective sample size of 2800 (calculated by dividing total patients by design effect = 60 × 700 = 42,000; 42,000/15 = 2800). A sample size of 2800 patients across all four study groups will allow us to detect a 5 % decrease in testing from 20 to 15 % between any two groups with 94 % power. A total of 15 general practices will be randomly allocated to each of the four study arms.

### Statistical analysis

Analyses will be according to intention to treat. The effect of removal of incentives and removal of feedback on chlamydia testing rates will be investigated by comparing the study groups as shown in the table below (Table 1). We will also investigate the effect of each of the two interventions on chlamydia testing rates as main effects exploiting the factorial nature of the design. Thus, the effect of removing financial incentives will be assessed by comparing randomised groups 2 and 3 with groups 1 and 4. Similarly, the effect of removing audit plus feedback will be assessed by comparing groups 1 and 3 with groups 2 and 4. The advantage of a factorial design is that it includes all patients in assessing the effectiveness of the two separate interventions, maximising the power of the study. However, this is based on the assumption that the effect of the two interventions is at least additive, and that there is no negative interaction between them, which will be tested. Formal statistical comparisons will be based on generalised mixed models that can account for cluster (general practice), GP and patient variability. Generalised estimating equation (GEE) approaches, with robust standard errors, will be adopted using STATA software. Hierarchical logistic regression models will be used to examine the impact of each of the two interventions over time on the proportion of patients who have a chlamydia test during the follow-up. Analyses will be adjusted for the chlamydia testing rate at each general practice immediately prior to commencing ACCEPt-able.

**Table 1:** Comparison of groups for analysis

Comparison	Removal of audit and feedback—continue financial incentives only	Removal of financial incentives—continue audit plus feedback only	Removal of financial incentives and audit plus feedback	Continue financial incentives and audit plus feedback
	Group 1	Group 2	Group 3	Group 4
Removal of financial incentives versus other	Groups 2 and 3 versus groups 1 and 4			
Removal of audit and feedback versus other	Groups 1 and 3 versus groups 2 and 4			

### Interviews with GPs and general practice staff

To evaluate the views and acceptability of incentive payments and external audit plus feedback, we will conduct semi-structured qualitative interviews with a sample of about 28 GPs (7 from each study arm) and 20 other staff including nurses and practice managers (5 from each study arm) towards the end of the ACCEPt-able intervention period. Recruitment numbers will be dependent upon reaching saturation of themes arising from iterative data analysis. All interviews will be conducted by telephone. Participants will be reimbursed for each interview ($AU 100 for GPs; $AU 40 for practice nurses/managers). The use of different sources of information (GPs, nurses, practice managers) is consistent with best practice in qualitative research (i.e. “triangulation” methods). The interview will cover (i) views and acceptability of audit plus feedback and incentive payments and their removal; (ii) perceived facilitators and barriers to preventive care activities; (iii) impacts of the trial on the general practice; and (iv) any contextual factors that may impact on the general practice’s workload and patient mix. Interviews will be tape-recorded and transcribed verbatim. After multiple readings of the text, data will be coded utilising a qualitative data software package. Consensus on main themes and sub-themes will be reached by multiple coders assigned to each transcription. Analyses will explore the acceptability of financial incentives and audit plus feedback and identify any barriers and facilitators to chlamydia testing in general practice.

### Cost-consequence evaluation

A cost-consequence analysis comparing costs and consequences in the scenarios for continuation/discontinuation of financial incentives and audit plus feedback activities will be undertaken. Costs (including incentives, travel, time) and consequences in the following scenarios will be compared: (i) financial incentives continue but audit plus feedback activities are removed; (ii) financial incentives are removed but audit plus feedback activities continue; (iii) both financial incentives and audit plus feedback activities are removed; and (iv) financial incentives and audit plus feedback activities continue. The consequence to be considered in each scenario is the proportion of people tested in the target population.

### Trial status

The trial has been registered on the Australian and New Zealand Clinical Trials Registry (ACTRN12614000595617). A total of 59 general practices have been recruited to ACCEPt-able with only one eligible practice refusing to participate (98 % response rate). The intervention period will be complete on 2017 with trial results due late 2017.

## Discussion

This will be the first RCT to examine the impact of removal of financial incentive payments and audit and feedback on the chlamydia testing behaviour of GPs. As both the Australian and UK governments act to reduce payments or raise the threshold to be eligible for payments, we urgently need robust epidemiological data assessing the potential impact this might have on provider performance and patient outcomes. Further, audit plus feedback are key components of managing quality improvement in general practice in Australia and the UK [4, 5], and while research suggests that it can be effective in modifying provider behaviour [6], there is no RCT evidence available about the impact its removal may have on GP activity.

The interactions between intrinsic motivation of doctors and extrinsic rewards such as financial remuneration are complex. It is difficult to judge the point at which incentivized behaviours become normalised within standard practice, rendering financial incentives superfluous. A recently published study examined the impact of removing financial incentives on provider behaviour in the Kaiser Permanente health plan in the USA. This observational study found that when financial incentives were removed from two indicators, there was a small decrease of about 3 % per year for screening for diabetic retinopathy and 1.6 % per year for cervical cancer screening [13]. A more recent analysis of observational data examined the effect of withdrawing incentives for eight clinical quality indicators from the QOF in the UK including influenza immunisation, lithium treatment and blood pressure, cholesterol and blood glucose monitoring. This study found that mean levels of performance were generally stable, in both the short and long term; however, there was a small but statistically significant drop in influenza immunisations [25].

This trial has several strengths. Firstly, a factorial RCT design is the most powerful and efficient method to investigate the impact of removing financial incentives and audit plus feedback on GP behaviour. In addition to the effects of each treatment, factorial design provides some data on the interaction that may exist between two interventions. Hence, we will have evidence about whether the effect of the removal of incentives varies according to whether or not practices receive audit plus feedback as well. Secondly, the inclusion of qualitative interviews will collect valuable data about the perceived importance of incentive payments and audit and feedback to general practice staff. These qualitative data will be assessed alongside the trial results, providing greater insight to the RCT results. Finally, the cost-consequence analysis also strengthens this trial. This trial will gather evidence to inform future policy on primary care reform, and as part of this, the cost of the interventions investigated and the outcomes associated with each intervention are of paramount importance to decision makers. A cost-consequence analysis involves itemising the different intervention components and their costs and the outcomes (chlamydia testing rate) for each trial group. We have selected this form of analysis because, unlike a cost-effectiveness analysis, the cost-consequence approach clearly lists the costs and outcomes associated with each intervention enabling decision makers to see the actual data. Unfortunately, one clinic refused to participate in the trial. However, this has negligible impact on our statistical power as we will still have 93 % power to detect a drop in chlamydia testing rates from 20 to 15 % between any two groups.

### Conclusion

While annual chlamydia screening is a key preventive health activity for young adults in Australia and the UK [18, 20], the results of this trial will have preventive health implications well beyond the content area of chlamydia. Incentive payments and audit plus feedback are already used for several preventive health activities in primary care in Australia and the UK including asthma management, cervical cancer screening and cardiovascular risk assessment. Our results will be readily transferable to these other preventive health activities. This trial is particularly timely as we will increase the understanding of the impact of financial incentives and audit and feedback on primary care provider behaviour when governments are looking for opportunities to control healthcare budgets and maximise clinical outcomes for money spent.

## Abbreviations

ACCEPt: Australia Chlamydia Control Effectiveness Pilot
GP: General practitioner
P4P: Pay for performance
QOF: Quality and outcomes framework
